# Niclosamide Suppresses Cancer Cell Growth By Inducing Wnt Co-Receptor LRP6 Degradation and Inhibiting the Wnt/β-Catenin Pathway

**DOI:** 10.1371/journal.pone.0029290

**Published:** 2011-12-16

**Authors:** Wenyan Lu, Cuihong Lin, Michael J. Roberts, William R. Waud, Gary A. Piazza, Yonghe Li

**Affiliations:** 1 Drug Discovery Division, Department of Biochemistry and Molecular Biology, Southern Research Institute, Birmingham, Alabama, United States of America; 2 Drug Development Division, Department of Cell Biology and Immunology, Southern Research Institute, Birmingham, Alabama, United States of America; 3 Drug Development Division, Department of Cancer Therapeutics, Southern Research Institute, Birmingham, Alabama, United States of America; 4 Mitchell Cancer Institute, University of South Alabama, Mobile, Alabama, United States of America; Medical College of Georgia, United States of America

## Abstract

The Wnt/β-catenin signaling pathway is important for tumor initiation and progression. The low density lipoprotein receptor-related protein-6 (LRP6) is an essential Wnt co-receptor for Wnt/β-catenin signaling and represents a promising anticancer target. Recently, the antihelminthic drug, niclosamide was found to inhibit Wnt/β-catenin signaling, although the mechanism was not well defined. We found that niclosamide was able to suppress LRP6 expression and phosphorylation, block Wnt3A-induced β-catenin accumulation, and inhibit Wnt/β-catenin signaling in HEK293 cells. Furthermore, the inhibitory effects of niclosamide on LRP6 expression/phosphorylation and Wnt/β-catenin signaling were conformed in human prostate PC-3 and DU145 and breast MDA-MB-231 and T-47D cancer cells. Moreover, we showed that the mechanism by which niclosamide suppressed LRP6 resulted from increased degradation as evident by a shorter half-life. Finally, we demonstrated that niclosamide was able to induce cancer cell apoptosis, and displayed excellent anticancer activity with IC_50_ values less than 1 µM for prostate PC-3 and DU145 and breast MDA-MB-231 and T-47D cancer cells. The IC_50_ values are comparable to those shown to suppress the activities of Wnt/β-catenin signaling in prostate and breast cancer cells. Our data indicate that niclosamide is a unique small molecule Wnt/β-catenin signaling inhibitor targeting the Wnt co-receptor LRP6 on the cell surface, and that niclosamide has a potential to be developed a novel chemopreventive or therapeutic agent for human prostate and breast cancer.

## Introduction

Wnt/β-catenin signaling plays an important role in embryonic development and can lead to tumor formation when aberrantly activated. A hallmark of the Wnt/β-catenin signaling activation is the stabilization of cytosolic β-catenin, which enters the nucleus to activate Wnt target genes by binding transcription factors of the T-cell factor/lymphoid enhancing factor (TCF/LEF) family [1], [2]. In the absence of Wnt ligands, β-catenin levels are efficiently regulated by a supramolecular complex containing adenomatous polyposis coli (APC), axin, and glycogen synthetase kinase 3β (GSK3β). This complex promotes phosphorylation of β-catenin by casein kinase 1 (Ck1) and GSK3β. Phosphorylated β-catenin becomes multi-ubiquitinated (Ub) and degraded by the 26S proteasome. The action of this complex is inhibited upon the binding of Wnt to its receptors on the cell surface [1], [2]. A variety of Wnt/β-catenin target genes have been identified, including those that regulate cell proliferation and apoptosis, thus mediating cancer initiation and progression [3]–[5]. Compelling evidence has indicated that there is an abnormal up-regulation of this pathway in tumorigenesis of many types of cancer, and that disruption of Wnt/β-catenin signaling represents a great opportunity to develop novel drugs for cancer chemoprevention and therapy [6]–[8].

Experiments performed in drosophila [9], xenopus [10] and mice [11] demonstrated that the low-density lipoprotein receptor-related protein 5 (LRP5)/LRP6 (termed arrow in drosophila) acts as a co-receptor for Wnt ligands, which interact with both the seven transmembrane receptor of the Frizzled (Fz) family and LRP5/6 to activate the canonical Wnt signaling pathway. The cytoplasmic tails of LRP5/6, upon receptor activation by Wnt ligand, are phosphorylated, and recruit the cytosolic scaffold protein axin to the membrane. LRP6 is expressed in human cancer cell lines and up-regulated in human malignant tissues [12]–[16]. Studies in the past years have demonstrated that LRP6 is a promising therapeutic target for the development of novel anticancer drugs [6]–[8], [15], [17], [18].

Niclosamide (trade name Niclocide) is a teniacide in the antihelmintic family that is especially effective against cestodes, which infects humans. Niclosamide has been FDA approved for such indications and has been used in humans for nearly 50 years [19]–[21]. It is believed that niclosamide inhibits oxidative phosphorylation in the mitochondria of cestodes; an aerobic metabolism, on which many cestodes are dependent. Recently, it has been demonstrated that niclosamide can downregulate cytosolic β-catenin expression to inhibit Wnt/β-catenin signaling [22], [23], and suppress colorectal cancer growth and metastasis [23], [24]. Since the mechanism was not well defined, we further studied this. We demonstrated for the first time that niclosamide can inhibit Wnt/β-catenin signaling by inducing LRP6 degradation, and that this activity is closely associated with its antiproliferative and apoptosis inducing activity.

## Results

### Niclosamide blocks Wnt/β-catenin signaling induced by Wnt3A and LRP6 by suppressing LRP6 expression in HEK293 cells

**Figure 1:**
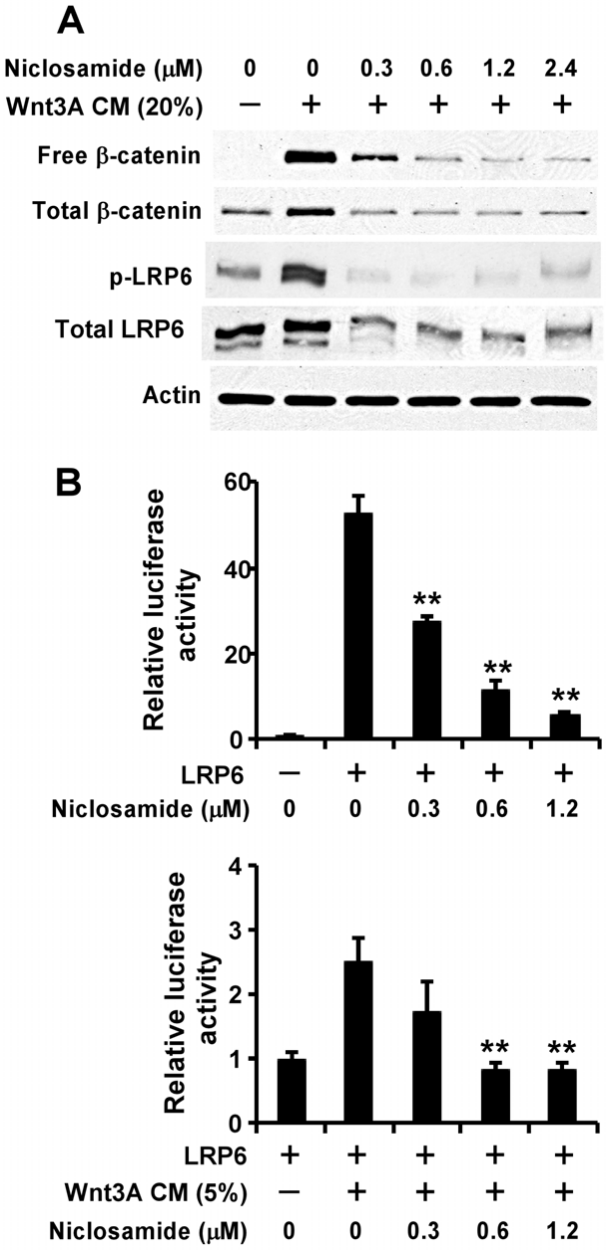
Effects of niclosamide on Wnt3A and LRP6-induced Wnt/β signaling in HEK293 cells. (A) HEK293 cells in 6-well plates were treated with Wnt3A CM (20%) and niclosamide at indicated concentrations for 24 h. The levels of cytosolic free β-catenin, total cellular β-catenin, LRP6 and phospho-LRP6 (p-LRP6) were examined. All the samples were also probed with anti-actin antibody to verify equal loading. (B) HEK293 cells in 24-well plates were transiently transfected with LRP6 plasmid or the corresponding control vector, along with TOPFlash construct and β-galactosidase-expressing vector in each well. After being incubated for 24 h, cells were treated with niclosamide or niclosamide plus Wnt3A CM at indicated concentrations for 24 h. The luciferase activity was then measured 24 h later with normalization to the activity of the β-galactosidase. Values are averages of three determinations with the standard deviations indicated by error bars. **P<0.01 compared to the control cells without niclosamide treatment.

Uncomplexed cytosolic β-catenin (free β-catenin) is the active form of β-catenin that is translocated to the cell nucleus to activate transcription factors of the TCF/LEF family, leading to the transcription of Wnt target genes. To determine if niclosamide can inhibit Wnt/β-catenin signaling, HEK293 cells were treated for with Wnt3A conditioned medium (CM) in the presence of niclosamide or vehicle (DMSO). The levels of cytosolic free β-catenin and total cellular β-catenin were then examined by Western blotting. As shown in Figure 1A, niclosamide was able to block Wnt3A-induced cytosolic free β-catenin and total cellular β-catenin accumulation in HEK293 cells at concentrations as low as 0.6 µM (Figure 1A).

LRP6 is an essential Wnt co-receptor for the Wnt/β-catenin signaling pathway, and LRP6 phosphorylation is critical for Wnt/β-catenin signaling activation induced by Wnt proteins [25]–[28]. To explore the molecular mechanism underlying Wnt/β-catenin signaling inhibition by niclosamide, we examined LRP6 expression and phosphorylation after niclosamide treatment. As shown in Figure 1A, treatment of Wnt3A CM markedly induced endogenous LRP6 phosphorylation in HEK293 cells, which was abolished by niclosamide treatment. Importantly, we also found that the total cellular level of endogenous LRP6 was greatly decreased after niclosamide treatment in HEK293 cells (Figure 1A).

To confirm the inhibitory effect of niclosamide on LRP6 expression and phosphorylation, we performed a Wnt/β-catenin signaling reporter assay to test whether niclosamide is able to inhibit Wnt/β-catenin signal activation by LRP6 and Wnt3A. HEK293 cells were transiently transfected with LRP6 along with Wnt/β-catenin signaling reporter TOPFlash, and treated with Wnt3A CM and niclosamide. As shown in Figure 1B, LRP6 expression increased the TOPFlash activity in HEK293 cells, which was further enhanced when the cells were transfected with LRP6 and treated with Wnt3A CM (Figure 1B). Moreover, the increased TOPFlash activity induced by LRP6 or LRP6 plus Wnt3A CM was blocked by niclosamide (Figure 1B).

### Niclosamide suppresses LRP6 Expression and Phosphorylation and Blocks Wnt/β-catenin Signaling in Prostate and breast cancer cells

**Figure 2:**
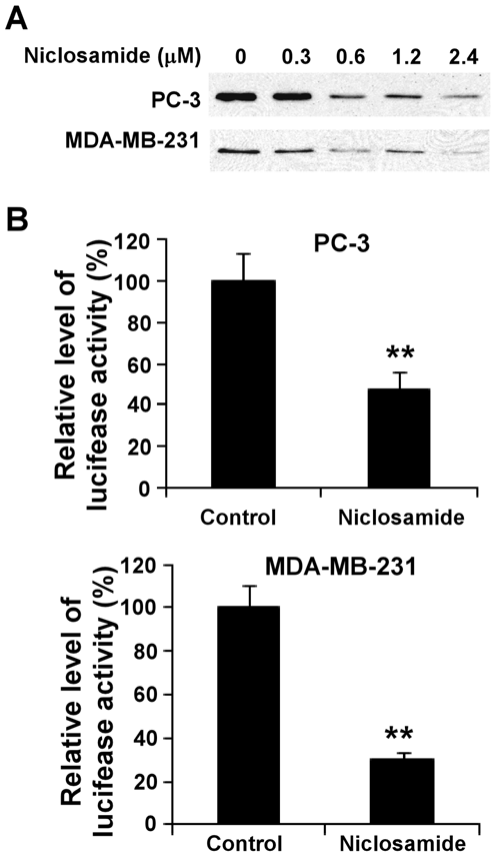
Effects of niclosamide on Wnt/β-catenin signaling in cancer cells. (A) Prostate cancer PC-3 and breast cancer MDA-MB-231 cells in 6-well plates were treated with niclosamide at the indicated concentrations for 24 h. The levels of cytosolic free β-catenin were then examined by GST-E-cadherin binding assay. (B) Prostate cancer PC-3 and breast cancer MDA-MB-231 cells in 24-well plates were transiently transfected with the TOPFlash luciferase construct and β-galactosidase-expressing vector in each well. After 24 h incubation, cells were treated with 2.4 µM niclosamide. The luciferase activity was then measured 24 h later with normalization to the activity of the β-galactosidase. Values are the average of triple determinations with the s.d. indicated by error bars. **P<0.01 compared to the control cells.

To determine if niclosamide can block Wnt/β-catenin signaling in cancer cells, we examined the level of cytosolic free β-catenin after niclosamide treatment. We found that cytosolic free β-catenin levels in human prostate PC-3 and breast MDA-MB-231 cancer cells were significantly reduced after niclosamide treatment (Figure 2A). Furthermore, when PC-3 and MDA-MB-231 cells were transiently transfected with the Wnt/β-catenin signaling reporter TOPFlash, we found that the niclosamide treatment greatly reduced the TOPFlash activity in PC-3 and MDA-MB-231 cells (Figure. 2B).

**Figure 3:**
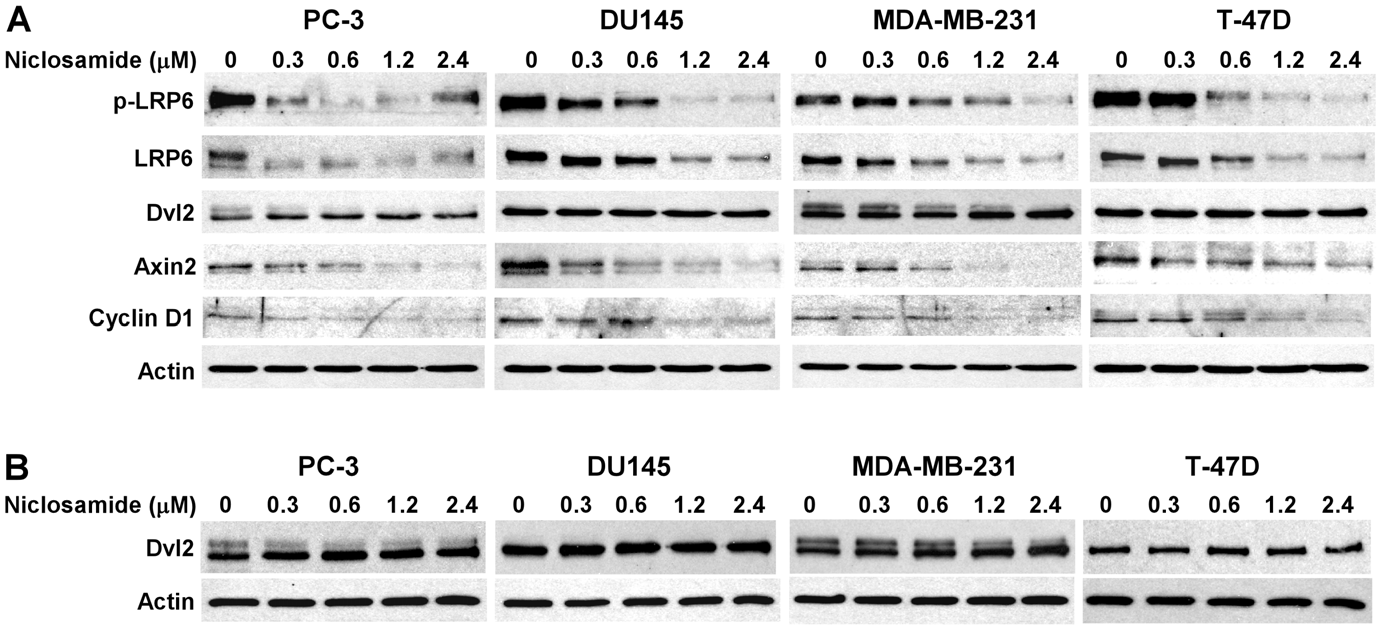
Effects of niclosamide on expression of LRP6, phosph-LRP6 (p-LRP6), Dvl2, Axin2 and Cyclin D1 in cancer cells. (A) DU145 and PC3 prostate cancer cells and MDA-MB-231 and T-47D breast cancer cells in 6-well plates were treated with niclosamide at indicated concentrations for 24 h. The total cellular levels of LRP6, p-LRP6, Dvl2, Axin2 and Cyclin D1were then examined. All the samples were also probed with anti-actin antibody to verify equal loading. (B) PC-3 and DU145 prostate cancer cells and MDA-MB-231 and T-47D breast cancer cells in 6-well plates were treated with niclosamide at indicated concentrations for 24 h. The cytosolic levels of Dvl2 were then examined. All the samples were also probed with anti-actin antibody to verify equal loading.

It is well recognized that axin2 is a specific transcriptional target of the Wnt/β-catenin signaling pathway, and that the expression level of axin2 is the signature of the activation of Wnt/β-catenin signaling [29]–[32]. Cyclin D1 is a transcriptional target of Wnt/β-catenin signaling, and is critical for cancer cell proliferation [33], [34]. To determine the inhibitory effects of niclosamide on Wnt/β-catenin signaling in human cancer cells, we examined axin2 and cyclin D1 expression in human prostate PC-3 and DU145 and breast MDA-MB-231 and T-47D cancer cells. As shown in Figure 3, niclosamide significantly suppressed axin2 and cyclin D1 expression in all four cancer cell lines.

To determine if the inhibitory effect of niclosamide on Wnt/β-catenin signaling is related to LRP6 expression, we examined LRP6 expression and phosphorylation after niclosamide treatment. As shown in Figure 3A, niclosamide was able to suppress LRP6 expression and phosphorylation in all four cancer cell lines, indicating that niclosamide can repress Wnt/β-catenin signaling in breast and prostate cancer cells by suppressing LRP6 expression.

### Niclosamide Induces LRP6 Degradation

**Figure 4:**
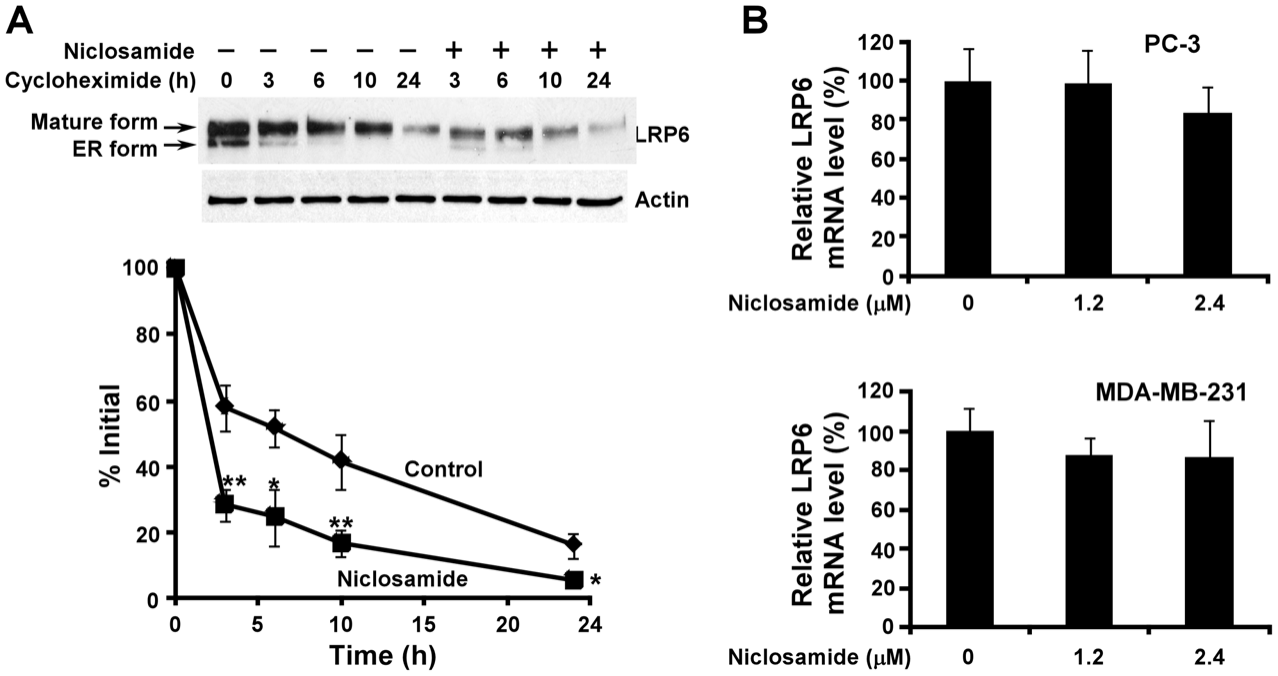
Effects of niclosamide on LRP6 expression and degradation. (A) PC-3 cells were incubated with 10 µg/ml of cycloheximide in the presence of Niclosamide (1.2 µM) or vehicle for 0, 3, 6, 10 or 24 h. Cells were then harvested, and the level of endogenous LRP6 was examined by Western blotting. Samples were also probed with anti-actin antibody to verify equal loading. The pixels for each band were measured, normalized and plotted. Data are mean values of three independent experiments with the SD values indicated by error bars. *P<0.05, **P<0.01 versus corresponding control value. (B) PC-3 and MDA-MB-231 cells in 6-well plates were treated with niclosamide for 24 h. The cells were then harvested and LRP6 mRNA levels were determined by real-time RT-PCR and normalized to the message levels of GAPDH mRNA. All the values are the average of triple determinations with the s.d. indicated by error bars.

To define the molecular mechanism underlying the effect of niclosamide on LRP6 protein level, we studied LRP6 turnover. PC-3 cells were treated with niclosamide at 1.2 µM for 0, 3, 6, 10 and 24 h in the presence of cycloheximide, a protein synthesis inhibitor. As shown in Figure 4, in the absence of niclosamide treatment, LRP6 was degraded with a half-life of about 6.9 h. Treatment with niclosamide significantly enhanced LRP6 turnover with a half-life of about 2.3 h (Figure 4A). This shortening of LRP6 half-life suggests that niclosamide can stimulate LRP6 degradation. To test whether niclosamide regulates LRP6 expression at the transcription level too, we examined LRP6 mRNA levels by real-time RT-PCR. As shown in Figure 4B, LRP6 mRNA levels were not significantly changed after niclosamide treatment in PC-3 and MDA-MB-231 cells. Therefore, niclosamide-induced LRP6 suppression is mainly due to the enhanced LRP6 degradation.

### Niclosamide Has No Effect on Dishevelled-2 (Dvl2) Expression in Prostate and Breast Cancer Cells

It has been reported that niclosamide downregulates components of the Wnt pathway, specifically cytosolic Dvl2 expression, resulting in diminished downstream Wnt/β-catenin signaling in colorectal cancers [23]. To test whether Dvl2 is also involved in niclosamide-induced Wnt/β-catenin signaling inhibition in prostate and breast cancer cells, we examined both total cellular and cytosolic levels of Dvl2 expression. Unexpectedly, we found that niclosamide had no effect on both total cellular and cytosolic Dvl2 expression in human prostate PC-3 and DU145 and breast MDA-MB-231 and T-47D cancer cells (Figure 3), while LRP6 expression was greatly suppressed at the same treatment (Figure 3A). These results indicate that LRP6 downregulation is the key mechanism underlying niclosamide-induced Wnt/β-catenin signaling inhibition in human prostate PC-3 and DU145 and breast MDA-MB-231 and T-47D cancer cells.

### Niclosamide Induces Prostate and Breast Cancer Cell Apoptosis and Inhibits Cancer Cell Proliferation

**Figure 5:**
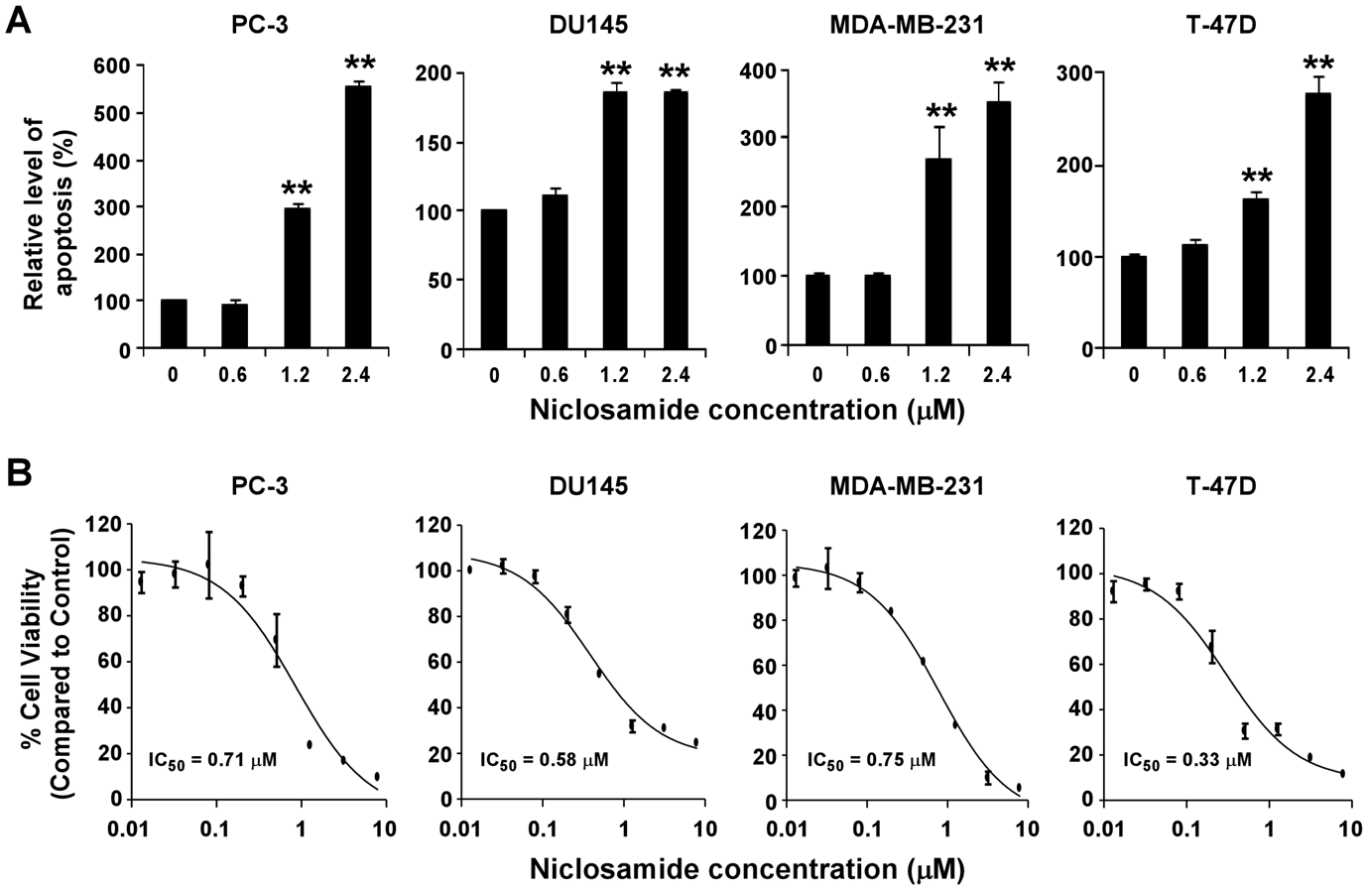
Effects of niclosamide on cancer cell apoptosis and proliferation. (A) Cancer cells were treated with niclosamide at the indicated concentrations for 24 h. Floating and attached cells were combined for apoptosis detection by the Cell Death ELISA kit from Roche Diagnostics Corporation for histone-associated DNA fragments as described in Materials and Methods. (B) Cancer cells in 96-well plates were treated with niclosamide for 72 h. Cell viability was measured by the Cell Titer Glo Assay system. All the values are the average of triplicate determinations with the s.d. indicated by error bars. **P<0.01 versus corresponding control value.

The Wnt/β-catenin pathway is important for cancer cell apoptosis [35], [36]. Having established that niclosamide is a potent inhibitor of Wnt/β-catenin signaling in prostate and breast cancer cells, we then tested whether niclosamide is able to induce apoptosis. As shown in Figure 5A, exposure to niclosamide at 1.2 and 2.4 µM for 24 h significantly induced apoptotic DNA fragmentation in prostate PC-3 and DU145 and breast MDA-MB-231 and T-47D cancer cells.

Given that niclosamide can block Wnt/β-catenin signaling in cancer cells and can induce apoptosis, we next examined the effect of niclosamide on cancer cell proliferation. As shown in Figure 5B, niclosamide inhibited cancer cell proliferation with IC_50_ values less than 1 µM for all four tested cell lines. The IC_50_ values are comparable to those shown to suppress the activities of Wnt/β-catenin signaling in prostate and breast cancer cells.

## Discussion

Compelling evidence indicates that there is an abnormal up-regulation of the Wnt/β-catenin pathway in tumorigenesis of many types of cancer. While genetic mutations of certain components of the Wnt/β-catenin pathway, such as *APC* and *CTNNB1*, are significant contributing factors for colorectal cancers, they are typically not the predominate mechanism associated with many other types of cancer such as breast and prostate cancers. Instead, it appears that dysregulation of Wnt/β-catenin signaling on the cell surface leads to aberrant activation of this pathway in these types of cancer [3]–[5],[37]. Therefore, targeted inhibition of Wnt/β-catenin signaling at the cell surface is a rational and promising new approach for the cancer therapy.

Wnt/β-catenin signaling plays an important role in mammary gland development and carcinogenesis [4]. Triple negative breast cancer (TNBC; ER, PR, and HER2-negative breast cancer) is one of the most difficult subtypes of breast cancer to treat due to a lack of a targeted therapy. Studies have demonstrated that Wnt/β-catenin signaling activation is preferentially found in TNBC and is associated with a poor clinical outcome [38]. Basal-like breast cancer (BLBC) shares many overlapping features with BLBC. Several studies have demonstrated that LRP6 is up-regulated in human TNBC or BLBC [14]–[16]. In mice, mammary gland development and MMTV-Wnt1-induced mammary tumorigenesis were delayed in LRP6^+/-^ mice [14], while MMTV-LRP6 transgenic mice developed hyperplasia in their mammary glands due to LRP6-mediated Wnt/β-catenin signaling [39]. Transcriptional knockdown of LRP6 in TNBC MDA-MB-231 cells significantly decreased Wnt/β-catenin signaling, cell proliferation, and tumor growth *in vivo* [15]. In addition, it has been demonstrated that specific LRP6 antibodies were able to block MMTV-Wnt1 or MMTV-Wnt3 xenografts *in vivo* [17], [18], and that recombinant Mesd protein, an LRP6 antagonist, markedly suppressed tumor growth in MMTV-Wnt1 xenograft models [15]. Moreover, salinomycin, a breast cancer stem cell killer [40], was recently demonstrated to be an inhibitor of Wnt/β-catenin signaling by inducing LRP6 degradation [41]. In the present study, we demonstrated that niclosamide suppressed LRP6 expression in TNBC MDA-MB-231 cells and ER-positive breast cancer T-47D cells, and inhibited breast cancer cell proliferation with IC_50_ values less than 1 µM. Our results support the concept that LRP6 is a promising therapeutic target for breast cancer including TNBC.

Accumulated evidence has demonstrated a significant role for Wnt/β-catenin signaling in the development and progression of human prostate cancer [3]. Although it is unclear whether LRP6 is up-regulated in prostate cancer, we have recently demonstrated that the LRP6 antagonist Mesd markedly inhibited Wnt/β-catenin signaling in prostate cancer PC-3 cells, and suppressed PC-3 cell proliferation *in vitro* and tumor growth *in vivo* [42], [43]. In the present study, we found niclosamide inhibited the proliferation of prostate cancer PC-3 and DU145 cells with IC_50_ values less than 1 µM, which are comparable to those shown to suppress LRP6 expression and the activities of Wnt/β-catenin signaling in prostate cancer cells. Together, these findings suggest that LRP6 is a potential therapeutic target for prostate cancer.

Mutations in *APC* and *CTNNB1* are two major factors for Wnt/β-catenin signaling activation in colorectal cancers, however recent studies indicate that the levels of Wnt/β-catenin signaling in colorectal cancer cells could be modulated on the cell surface [44]–[48]. In particular, Ueno *et al.* demonstrated that Wnt receptor frizzled-7 activates the Wnt/β-catenin pathway in colorectal cancer cells despite the presence of *APC* or *CTNNB1* mutation and that frizzled-7-siRNA may be used as a therapeutic reagent for colorectal cancer [48]. Shi *et al*. reported that siRNA silencing of Wnt-2, which is well known for its overexpression in colorectal cancer, inhibited Wnt/β-catenin signaling and induced apoptosis in human colorectal cancer cells containing downstream mutations [47]. The secreted frizzled-related protein (SFRP) family and Wnt inhibitory factor-1 (WIF-1) are secreted Wnt/β-catenin signaling antagonists that bind to Wnt ligands, and interfere with Wnt-receptor interaction. It has been reported that loss of SFRP family expression was associated with promoter hypermethylation in colorectal cancer [44], and that restoration of SFRP function in colorectal cancer cells attenuates Wnt/β-catenin signaling even in the presence of downstream mutations [45]. Similarly, He *et al.* reported that loss of WIF-1 expression was associated with promoter hypermethylation in colorectal cancer, and that restoration of WIF-1 function, Wnt-1 siRNA, or a monoclonal anti-Wnt-1 antibody induces significant apoptosis in colorectal cancer cells which contain downstream mutations and expressing Wnt-1 [46]. Very recently, it has been reported that niclosamide was able to inhibit Wnt/β-catenin signaling in colorectal cancer cells, inhibited colorectal cancer growth and S100A4-mediated metastatic colorectal cancer [23], [24]. The calcium-binding protein S100A4 is a target gene of the Wnt/β-catenin pathway [49]. Future studies are required to address the importance of LRP6 in colorectal cancer development and progression.

The development of FDA approved drugs that are used for other indications is an attractive strategy for developing new drugs for cancer chemoprevention or therapy given their proven safety record. Disruption of Wnt/β-catenin signaling represents a great opportunity to determine if FDA approved drugs might have potential anticancer efficacy [6]–[8]. Niclosamide is a salicyclamide derivative and the drug of choice for the treatment of most tapeworm infection. Niclosamide exerts its antiparasitic activity in the intestinal lumen and is poorly absorbed from the gastrointestinal tract [19]–[21]. However, in mice implanted with human colorectal cancer xenografts, orally administered niclosamide was well tolerated, achieved plasma and tumor levels associated with biologic activity and led to tumor control [23]. Furthermore, niclosamide has no significant toxicity against non-cancer cells *in vitro* and displays no obvious side effects in niclosamide-treated mice [23]. We demonstrate herein that niclosamide is a potent Wnt/β-catenin signaling inhibitor by inducing LRP6 degradation in cancer cells, and displays an excellent anticancer activity *in vitro*. Therefore, as a FDA approved antihelminthic drug, niclosamide will be an interesting comoound to be optimized to increase its bioavailability and to be tested for its *in vivo* cancer preventive and therapeutic roles in the future.

## Materials and Methods

### Materials

Niclosamide was purchased from Sigma. Polyclonal anti-phospho-LRP6 (Ser1490) and anti-Dvl2 and monoclonal anti-Axin2 were purchased from Cell Signaling Technology. Monoclonal anti-LRP6 was from Santa Cruz Biotechnology. Polyclonal rabbit anti-Cyclin D1 was from Chemicon International. Monoclonal anti-β-catenin was from BD Biosciences. Monoclonal anti-actin was from Sigma. Peroxidase labeled anti-mouse antibody and ECL system were purchased from Amersham Life Science. Plasmid pCS-Myc-hLRP6 containing the full-length human LRP6 cDNA was from Dr. Christof Niehrs (Deutsches Krebsforschungszentrum, Heidelberg, Germany). Plasmid Glutathione S-transferase (GST)-E-cadherin was kindly provided by Dr. Gail Johnson (University of Rochester, NY). The TOPFlash luciferase construct was from Upstate Biotechnology. The β-galactosidase-expressing vector, the luciferase assay system and the β-galactosidase assay system were purchased from Promega. Tissue culture media, fetal bovine serum (FBS), and plastic-ware were obtained from Life Technologies, Inc. Proteinase inhibitor cocktail Complete™ was obtained from Boehringer Mannheim. All cell lines were obtained from ATCC and were cultured in RPMI-1640 medium containing 10% fetal bovine serum, 2 mM of L-glutamine, 100 units/ml of penicillin, and 100 µg/ml of streptomycin. Cells were grown in antibiotic-free medium for cell proliferation assays. Cell Titer Glo assay systems were from Promega. Cell death detection ELISA kit was purchased from Roche Diagnostics Corporation.

### Western blotting

Cells in 6-well plates were lysed in 0.5 ml of lysis buffer (phosphate-buffered saline containing 1% Triton X-100 and 1 mM PMSF) at 4°C for 10 min. Equal quantities of protein were subjected to SDS-PAGE under reducing conditions. Following transfer to immobilon-P transfer membrane, successive incubations with a primary antibody, and a horseradish peroxidase-conjugated secondary antibody were carried out for 60–120 min at room temperature. The immunoreactive proteins were then detected using the ECL system. Films showing immunoreactive bands were scanned by Hp Scanjet 5590.

### Cytosolic free β-catenin analysis with GST-E-cadherin binding assay

The GST-E-cadherin binding assay was carried out exactly as previously described [50]. Uncomplexed cytosolic free β-catenin present in 100 µg of total cell lysate was subjected to SDS-PAGE and detected using the monoclonal antibody to β-catenin.

### Real-time RT-PCR

Total RNA was isolated from cell cultures using RNA-Bee (Tel-Test), and first-strand cDNA synthesis was performed using ProSTARTM Ultro HF RT-PCR Kit (Strategene) primed with oligo(dT) primer in a 20 µl reaction mixture containing 1 µg total RNA. For analysis of LRP6 mRNA levels, real-time RT-PCR was performed with the specific LRP6 and GAPDH primers purchased from SABioscience/Qiagene. The LRP6 mRNA level was normalized to the GAPDH mRNA level.

### Analysisof cytosolic Dvl2 expression

Cytosolic Dvl2 expression was examined with the method as described for elsewhere [23]. Briefly, cells were lysed with hypotonic lysis buffer (consisting of 10 mM Tris-HCl, pH 7.4 and 0.2 mM MgCl2, supplemented with protease inhibitors, incubated on ice for 10 min, and homogenized using a tight-fitting glass dounce. Sucrose and EDTA were added to final concentrations of 0.25 M and 1 mM, respectively. Lysates were then centrifuged at 20,000×g for 1 h at 4°C. Supernatant representing cytosolic cell fraction was then diluted into SDS sample buffer, and Dvl2 expression was then examined by Western blotting.

### Luciferase reporter assay

Cancer cells were plated into 24-well plates. After overnight culture, the cells were transiently transfected with 0.1 µg of the TOPFlash luciferase construct (Upstate Biotechnology) and 0.1 µg of β-galactosidase-expressing vector (Promega, Madison, WI). After 24 h incubation, cells were treated with niclosamide. Cells were then lysed 24 h later and both luciferase and β-galactosidase activities were determined. The luciferase activity was normalized to the β-galactosidase activity.

### Apoptosis evaluation

Apoptosis was assessed with cell death detection ELISA kit purchased from Roche Diagnostics Corporation, Indianapolis. This assay detects oligonucleosomes released after gentle lysis of the cell. Cells were cultured in T25 flasks for the desired duration. The spent medium containing floating cells was saved and kept on ice. The adherent cells were collected by gentle trypsinization and were combined with the floaters for pelleting by centrifugation. After gentle lysis of the cells with the buffer provided with the detection kit, the cell lysate was used for the ELISA test. The results were normalized by the protein content obtained from parallel flasks with the cells being lysed using the buffer as described above for Western blotting.

### Cell proliferation assay

Cells were seeded into 96-well tissue culture treated microtiter plates at a density of 5000 cells/well in a total volume of 50 µl. After overnight incubation, the cells were treated with niclosamide for 72 h by adding 50 µl of 2X stock solutions to appropriate wells already containing 50 µl of cells and medium to expose cells to the final concentrations of niclosamide required. Cell viability was measured by the Cell Titer Glo Assay (Promega).

### Statistics

Statistical analyses were performed using Student's unpaired t-test. Data were presented as mean ± SD. Differences at P<0.05 were considered statistically significant.
